# Dose Optimization for Single-Channel Vaginal Cylinder High-Dose-Rate Brachytherapy: A Double Prescription Method for Patients With Endometrial Adenocarcinoma

**DOI:** 10.7759/cureus.26303

**Published:** 2022-06-24

**Authors:** Xingen Wu, Andrew McDonald, Sui Shen, Dennis Stanley, Richard Popple, Samuel Marcrom, Robert Kim

**Affiliations:** 1 Radiation Oncology, University of Alabama at Birmingham Heersink School of Medicine, Birmingham, USA

**Keywords:** dose prescription, endometrial adenocarcinoma, dose optimization, hdr brachytherapy, vaginal cylinder

## Abstract

Purpose

This study aimed to explore the relationship between applicator surface dose and 5 mm-depth dose and to optimize both locations simultaneously for three most used cylinder sizes (2.5, 3.0, and 3.5 cm in diameter) in treating patients with endometrial adenocarcinoma.

Materials and methods

A total of 216 plans were created for each dose level and applicator size. For each dose level, four plans were created with single or double prescription doses. For plans with double prescription doses, the dose constraints were applied to all those points on the surface and 5 mm depth and optimize the two sites simultaneously.

Results

A dose table between surface and 5 mm depth and its fifth order polynomial mapping functions were established for each applicator size, so any prescribed dose at one site can find the prescription dose on the other site in optimization on both locations. For plans with a 5 mm-depth prescription, the maximum dose on the surface can be reduced from 145% to 133% if the surface prescription dose is also used; for plans with surface dose prescription, the minimum dose and mean dose can be improved by 2% if 5 mm-depth dose prescription is also used in optimization.

Conclusion

Dose table and their mapping functions between surface prescription dose and their corresponding 5 mm-depth doses were created. A new optimization method that uses two prescription doses on both surface and 5 mm-depth sites was proposed to reduce the hot dose on the surface and improve the cold dose at 5 mm depth.

## Introduction

Endometrial cancer is the most common gynecological cancer among women in the United States. Surgery is the primary treatment for endometrial cancer. However, the most common local recurrence site has been in the upper vagina. Vaginal cylinder brachytherapy is as effective as pelvic radiation with lower toxicities at preventing vaginal recurrence for high-intermediate risk group of stage 1 endometrial carcinoma [1]. The American Brachytherapy Society (ABS) published detailed guidelines for endometrial high-dose-rate (HDR) brachytherapy, which include applicator selection, insertion techniques, target volume definition, dose fractionation, etc., and the vagina at risk needs proper coverage for 3-5 cm along the cylinder with the dose prescribed either at the applicator surface or at 5 mm depth from the surface [2]. Lots of research on HDR optimization has been published and a comprehensive review can be found in [3-5]. In this study, we compared the surface dose and 5 mm-depth dose and created a connection between these two sites. We analyzed the dose gradient of applicators with the three different sizes and proposed a new corresponding dose table between surface and 5 mm depth to make prescription doses on both sites possible in treatment planning. Based on the dose table, we also generated six mapping functions so any prescription dose at 5 mm depth can be found through the known prescription surface dose or from its mapping function. The same method can be applied to obtain the surface prescription dose through the known 5 mm-depth prescription dose. Later, we evaluated dose distribution at the surface and 5 mm depth from four different prescription methods.

## Materials and methods

**Figure 1:**
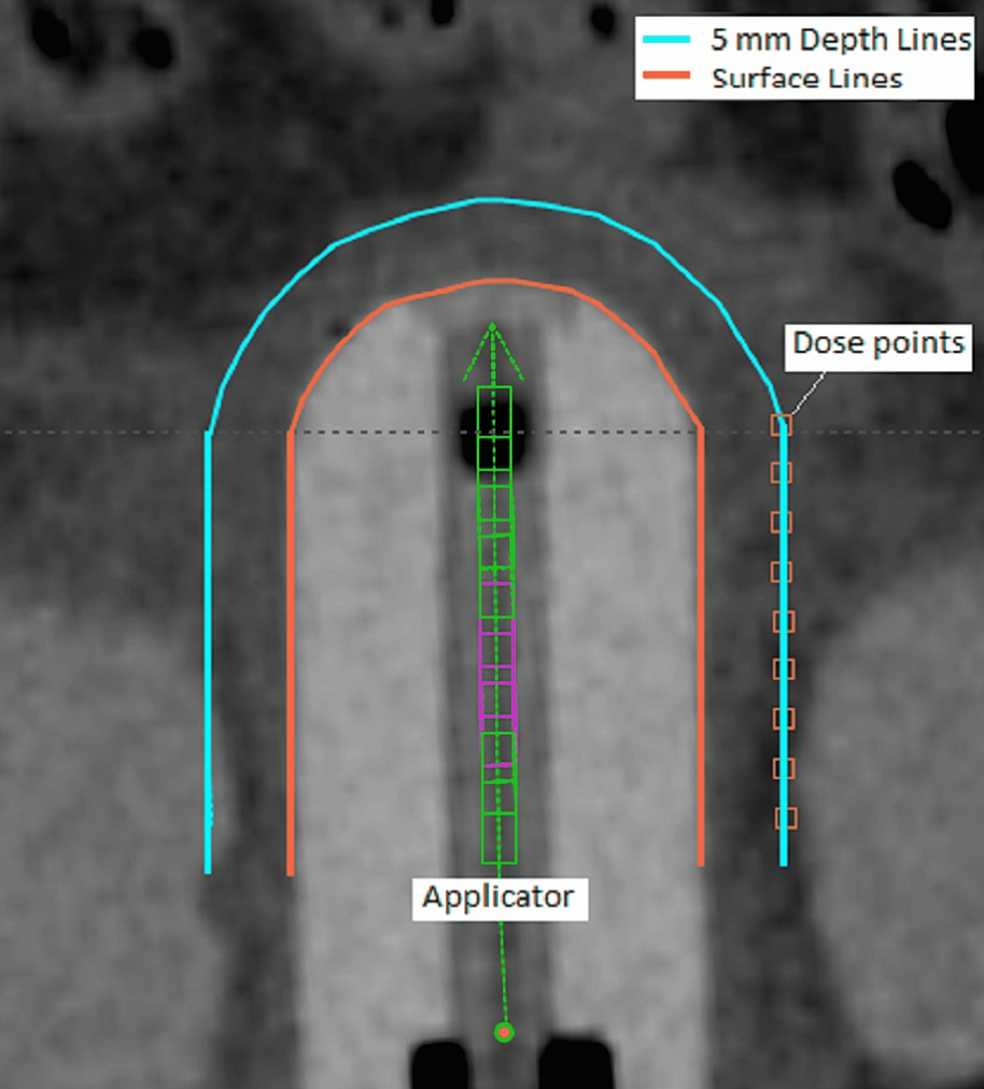
Single applicator and three surface reference lines (color: tomato) and three 5 mm-depth reference lines (color: cyan)

The cylinder applicator we used has a single channel (Figure 1). Before planning, three reference lines (left and right straight lines and one apex line) were created on the applicator surface and another three reference lines at 5 mm distance away from the surface. The source dwelling step size was always 5 mm. The dose points from these six lines can be used for dose constrains in volume optimization, which is VEGO TG-43 volume optimization in Varian’s Eclipse Brachytherapy planning system (Palo Alto, CA: Varian Medical System, Inc.) (Figure 1). We collected a total of nine dose levels for the three common cylinder sizes used in our department. For each prescription dose level, four plans were created for treatment planning.

If the primary prescription dose is at 5 mm depth, the dose constrains in the first plan "P1_5mm" are applied only to 5 mm depth. In the second plan "P2_5mm&Sur," the dose constraints are applied to dose points at 5 mm depth and on the surface simultaneously, and 5 mm-depth dose points are assigned to a higher priority (>100) and the surface dose points are assigned to a lower priority (<80). The dose constraints are similar to those in the external beam optimization; the doses of 98% target volume (dose points) are between ±50 cGy of the prescription doses. Dose constraints to the organs-at-risk (OAR), like rectum, bladder, small bowel, etc., were not considered as routine clinical practice in vaginal cylinder planning.

If the primary prescription dose is on the surface, the dose constraints in the third plan "P3_Sur" are applied only on the surface point doses. In the fourth plan "P4_Sur&5mm," the dose constraints are applied to dose points on the surface and 5 mm depth simultaneously, and the surface dose points are assigned to a higher priority (>100) and the 5 mm-depth points are assigned to a lower priority (<80).

Although P2_5mm&Sur and P4_Sur&5mm use the double prescription doses in the same plan, they use different priority strategies. In P2_5mm&Sur, the prescription dose at 5 mm depth is the primary and the dose points at this site are assigned a higher priority; in the P4_Sur&5mm plan, the above primary and priority are reversed. Even in the same higher or lower priority group, the priorities of the three lines are different due to the anisotropic effect of the source and cylinder construction. The apex line is usually assigned to a higher priority compared to the two straight lines and its priority is usually 10 ~ 50 higher (Figure 1). 

In all the optimization, the "smooth" parameter is assigned to the highest value, which is 300 to smooth out the dwelling time at each dwelling position to reduce the hot spot. The doses from both sites were normalized to their prescription dose respectively even in plans P1_5mm and P3_Sur which do not have the secondary prescription dose for easy comparison.

## Results

A total of 216 plans were created (9 dose levels x 4 plans/level x 3 Cylinder size x 2 patients/size) and half of them were analyzed and compared in dosimetric parameters. A table between surface dose and 5 mm-depth dose for prescription was generated after dose distribution analysis from these examples (Table 1). For each cylinder size, the first column shows the dose at 5 mm depth and the second column is the corresponding dose on the cylinder surface. 

**Table 1:** A prescription dose table between surface and 5 mm depth for three cylinder applicator sizes

2.5 cm cylinder		3.0 cm cylinder		3.5 cm cylinder	
5 mm depth (cGy)	Surface (cGy)	5 mm depth (cGy)	Surface (cGy)	5 mm depth (cGy)	Surface (cGy)
300	500	340	500	360	500
400	660	400	600	400	550
430	700	430	640	430	590
500	800	500	730	500	710
550	880	550	830	550	810
600	950	600	910	600	890
700	1100	700	1050	700	1000
800	1250	800	1200	800	1150
850	1350	850	1300	850	1250

The coefficients of three fifth order polynomial functions (y = a * x^5^ + b * x^4^ + c * x^3^ + d * x^2^ + e * x + f) for mapping 5 mm-depth dose to surface dose were obtained and another three functions for mapping in the reverse direction were listed in Table 2. The columns from two to four are the coefficients of the polynomial functions which are mapping any dose from 5 mm depth dose to the surface dose used for the dual prescriptions. The columns from five to seven are the opposite.

**Table 2:** Coefficients for the fifth polynomial mapping functions

Coefficients	From 5 mm to surface			From surface to 5 mm		
	2.5 cm	3.0 cm	3.5 cm	2.5 cm	3.0 cm	3.5 cm
a	0.004975	0.007740	-0.004073	-0.000316	-0.000461	0.000448
b	-0.140437	-0.219375	0.158500	0.014380	0.019668	-0.023634
c	1.550614	2.427377	-2.295163	-0.256824	-0.326899	0.472499
d	-8.351723	-13.101861	15.633410	2.245143	2.645898	-4.488110
e	23.370814	36.031033	-48.875896	-8.909707	-9.778320	20.930508
f	-21.646311	-35.645704	61.292976	15.522264	16.149188	-34.560913

**Figure 2:**
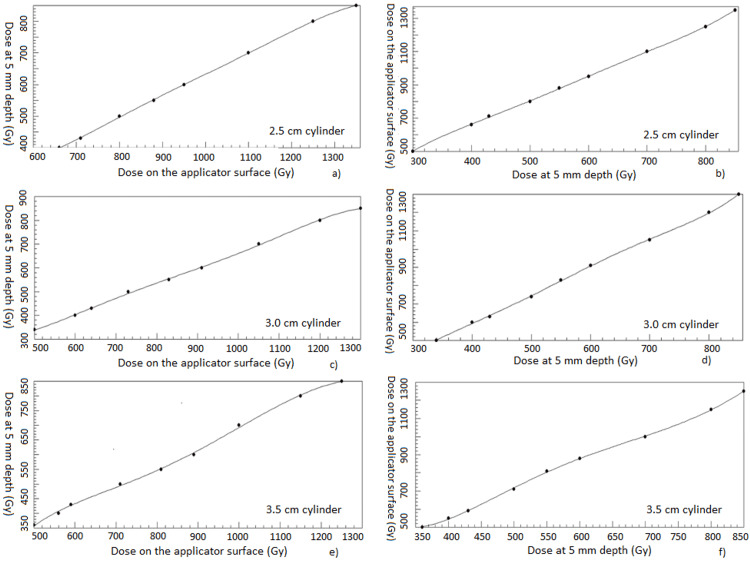
Six dose mapping functions between surface dose and 5 mm depth dose for three different cylinder applicator sizes (a) 2.5 cm cylinder dose mapping from surface to 5 mm depth; (b) 2.5 cm cylinder dose mapping from 5 mm depth to surface; (c) 3.0 cm cylinder dose mapping from surface to 5 mm depth; (d) 3.0 cm cylinder dose mapping from 5 mm depth to surface; (e) 3.5 cm cylinder dose mapping from surface to 5 mm depth; (f) 3.5 cm cylinder dose mapping from 5 mm depth to surface.

Figure 2 shows the mapping curves which can be used to find any prescription dose from one site to the other within the ranges.

**Figure 3:**
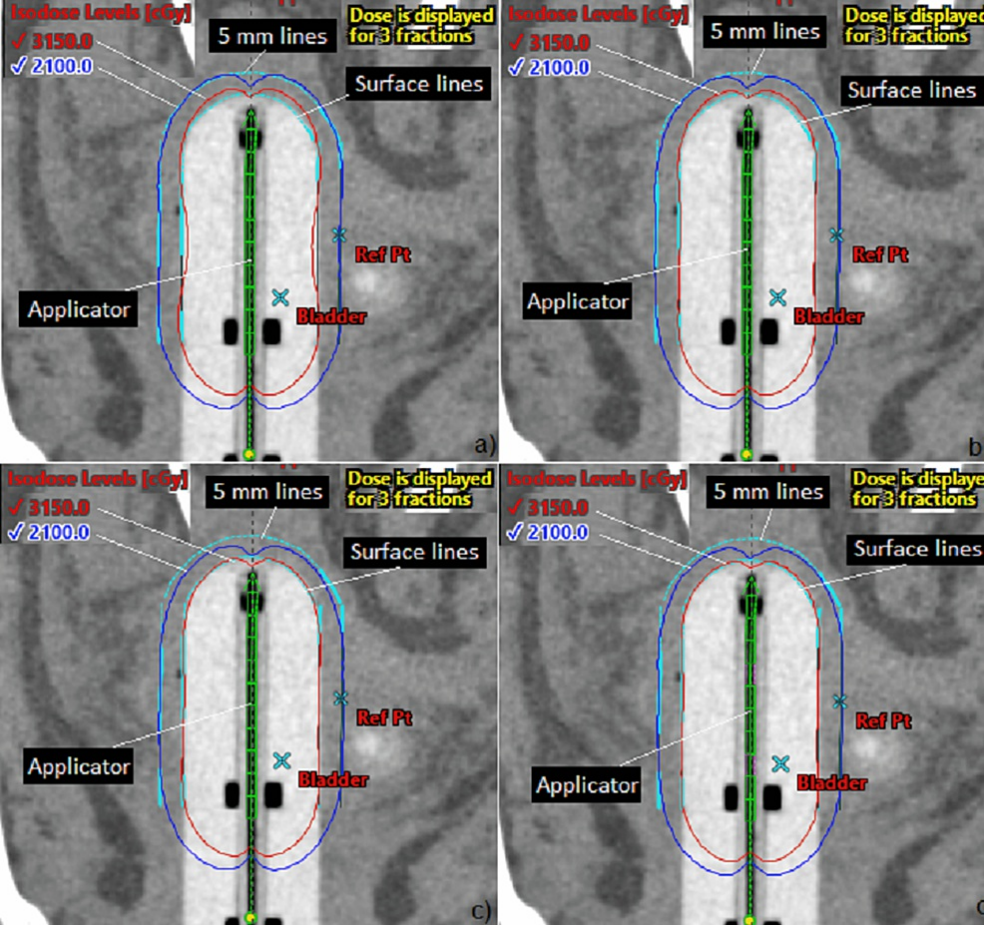
Dose distribution comparison among the four plans (a) P1_5mm; (b) P2_5mm&Sur; (c) P3_Sur; (d) P4_Sur&5mm. The two prescription doses are 2100 cGy (700 cGy per fraction) at 5 mm depth and 3150 cGy (1050 cGy per fraction) on the surface and the cylinder size is 3 cm. P1_5mm: Plan #1 prescribed only at 5 mm depth; P2_5mm&Sur: Plan #2 prescribed at 5 mm depth and on the surface simultaneously; P3_Sur: Plan #3 prescribed only on the surface; P4_Sur&5mm: Plan #4 prescribed on the surface and at 5 mm depth simultaneously.

Figure 3 shows the dose distribution on the sagittal plane in the four plans and the cylinder size is 3 cm. Two isodose lines, 100% (2100 cGy) and 150% (3150 cGy), are displayed in the images. The two prescription doses are 2100 cGy (700 cGy/fraction) at 5 mm depth and 3150 cGy (1050 cGy/fraction) on the surface (Figure 3). 

We also compared the maximum dose (DMax), minimum dose (DMin), and mean dose (DMean) of the 108 plans among the four prescription approaches (Table 3).

**Table 3:** Dosimetry comparison of surface dose and 5 mm depth dose from 108 plans along four prescription methods P1_5mm: Plan #1 prescribed only at 5 mm depth; P2_5mm&Sur: Plan #2 prescribed at 5 mm depth and on the surface simultaneously; P3_Sur: Plan #3 prescribed only on the surface; P4_Sur&5mm: Plan #4 prescribed on the surface and at 5 mm depth simultaneously. σ: standard deviation

	Surface dose			5 mm depth dose		
	Maximum (%)±σ	Minimum (%)±σ	Mean (%)±σ	Maximum (%)±σ	Minimum (%)±σ	Mean (%)±σ
P1_5mm	145.24±5.33	93.16±2.92	108.28±4.63	112.16±0.58	92.23±0.74	100.66±0.19
P2_5mm&Sur	133.40±3.81	94.32±2.45	105.70±3.53	105.53±1.66	90.83±0.84	98.80±1.22
P3_Sur	113.99±2.66	89.59±1.59	100.36±0.65	101.65±3.04	86.44±3.12	94.65±3.48
P4_Sur&5mm	117.16±3.15	92.08±2.68	101.64±1.24	101.85±2.08	87.94±2.49	95.82±2.74

The standard deviations (σ) were also calculated to show their variations. The maximum, minimum, and mean doses of surface are the maximum, minimum, and mean doses of all surface points from the left, right lines, and apex curves. The maximum, minimum, and mean doses of 5 mm-depth are the same.

## Discussion

The ABS recommends reporting doses at both the vaginal surface and at 5 mm vaginal mucosal depth. Also, the ABS recommends placing optimization points both at the apex and along the curved portion of the cylinder dome in addition to the lateral vaginal mucosa as shown in this dosimetry study. Our study offered a method to transfer from prescribing to 5 mm to prescribing to surface while still having the same 5 mm depth dose and a more uniform surface dose. Although some "rules of thumb" of doses between cylinder surface and 5 mm depth exist in the HDR brachytherapy community, they are not accurate enough for prescription. Our study showed the relationship between surface dose and 5 mm dose is nonlinear and that is the reason we chose the fifth order polynomial functions for mapping (Figure 2).

The most common early side effect of vaginal brachytherapy is vaginal irritation, dryness, discharge, and soreness. The common late side effects are vaginal itching, contact bleeding, shorting or narrow vagina, and dyspareunia due to microvascular damage. However, to our knowledge, there are no comparisons of side effects between vaginal surface and 5 mm-depth dose prescriptions. De Boer et al. reported long-term side effects which were treated with vaginal brachytherapy (VBT), 21 Gy in three fractions at 5 mm vs. external beam radiation therapy (EBRT), 46 Gy in 23 fractions in PORTEC-2 endometrial trial: bowel urgency 6.6% vs. 23.3%, fecal leakage 1.8% vs. 10.6%, urinary urgency 25.3% vs. 39.3% and no difference in sexual activity [6]. Patients with VBT arm experienced better social functioning, less bowel toxicity, and better quality of life. Vaginal dryness, shortening, or pain was not significantly different between the two treatment arms. In VBT arm, G3-G4 late vaginal toxicities were in less than 2% which consisted of slight atrophy, bleeding, and stenosis, VBT is very well tolerated even using different hypo-fractionated dose schedules.

Previous research almost covered every aspect from applicator size, tip space, dwelling step size, dose calculation models, optimization methods, etc., and their effects on dose distribution [6-12]. For example, Li et al. compared the three dose models: an isotropic and anisotropic dose calculation based on TG-43 formalism and the benchmark calculation using Monte-Carlo calculated dosimetry data [7]. Kim et al. studied the effect of the apex optimization line and found the use of this line is important, which can improve the cold dose at 5 mm depth, especially for their new vaginal cylinders which have thicker tops [8]. Optimization methods, classified as forward and inverse optimization, were also studied to compare the dose distribution, target coverage, and OAR sparing [9]. Li et al. studied the effects of the prescription depth, cylinder size, treatment length, tip space, and curved end on dose distribution and found the surface prescription has a more uniform dose at all depths in the target volume than 5 mm-depth prescription which creates large dose variations (50% ~ 70%) at the surface [10].

**Figure 4:**
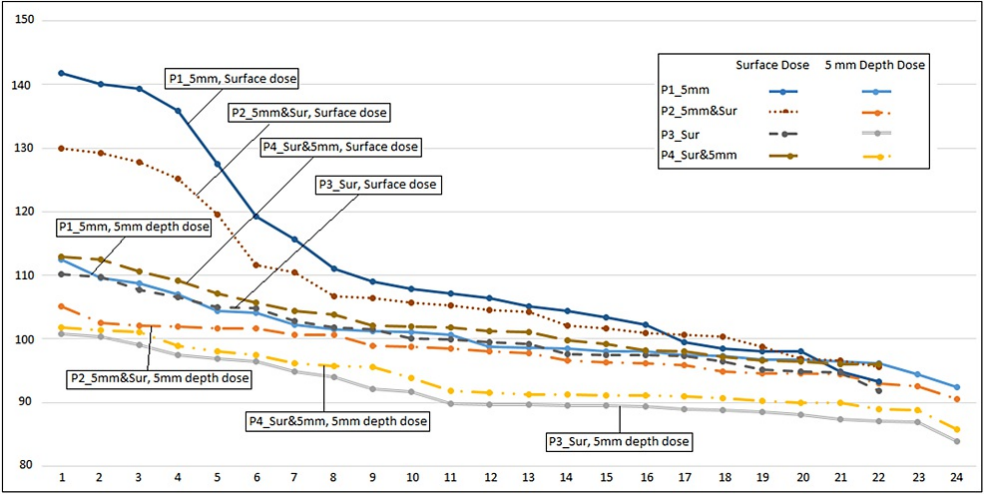
Dose profiles on the surface lines and at 5 mm depth lines sorted from highest to lowest in the four plans: 600 cGy was prescribed to 5 mm depth and 950 cGy to the surface with cylinder size 2.5 cm in diameter P1_5mm: Plan #1 prescribed only at 5 mm depth; P2_5mm&Sur: Plan #2 prescribed at 5 mm depth and on the surface simultaneously; P3_Sur: Plan #3 prescribed only on the surface; P4_Sur&5mm: Plan #4 prescribed on the surface and at 5 mm depth simultaneously

In our study, we proposed a new method: prescription doses to both sites. So optimizer can optimize dose distribution on surface and 5 mm depth simultaneously. The benefits are obvious: the doses in the whole treatment volume are more uniform. Figure 4 shows the point dose sorted from the highest to lowest on the lines, the hot dose points are usually located in the apex lines.

For dose prescriptions at 5 mm depth (P1_5mm & P2_5mm&Sur), the secondary surface dose prescription and optimization can reduce D_Max_ from 145.2% to 133.4%, while the dose distribution at 5 mm depth won’t change much. For dose prescriptions on the surface (P3_Sur & P4_Sur&5mm), the secondary 5 mm-depth dose prescription and optimization won’t change the hot doses on the surface much, but it improves the D_Min_ and D_Mean_ at both sites (Figure 3).

If we compared the four plans, the surface doses are much more uniform in P3_Sur and P4_Sur&5mm than in P1_5mm and P2_5mm&Sur. This conclusion is similar to one of the conclusions described in [9]. Our work further provides a solution on how to change the prescription at 5 mm depth to prescription on the surface. For those physicians who usually prescribe dose to 5 mm depth, the prescription dose table and the mapping functions can provide specific prescription doses on the surface, which greatly reduce the hot spots from 30~40% higher to around 15% higher (Table 1).

If we compare P2_5mm&Sur and P4_Sur&5mm, the two plans optimize dose points on both sites simultaneously but with different optimization priorities, the D_Max_ on the surface can be reduced from 33% higher to 15% higher while the other parameters are almost the same. In P2_5mm&Sur, the 5 mm-depth dose prescription is primary and surface prescription is secondary in optimization; in P4_Sur&5mm, the surface prescription is primary and the 5 mm-depth prescription is secondary. The secondary site can be used for dose comparison and reference. We normalized all point doses to their respective prescription dose to compare the dose uniformity even in P1_5mm and P3_Sur. This is different from the normalization method in Li et al.’s study in which a mean dose was used [8]. So the low doses at 5 mm depth in the plan P3_Sur and P4_Sur&5mm were not really cold dose. 

Among the four plans, P4_Sur&5mm has the highest quality and P3_Sur the second, P2_5mm&Sur the third and P1_5mm is the least if we use dose uniformity to evaluate these four plans (Figure 4). If higher dose is needed in the vaginal cuff (apex area), then the sequence will be reversed. 

In this study dose constraints to the regional OAR were not included in the optimization, as performed in routine clinical practice for vaginal cylinder applicator. This is because of the relatively lower energy of iridium-192 compared to the external beam, and the dose to the surrounding organs decreases very quickly from 5 mm prescription dose lines. So, we focus on the dosimetry and corresponding prescription doses between applicator surface and 5 mm depth. Due to specific features of fifth order polynomial function, our mapping functions are only for interpolation, not for extrapolation in the prescription dose range list in Table 1.

## Conclusions

A dose table for each applicator size from 2.5 cm to 3.5 cm was presented to illustrate the relationship between dose on the applicator surface and dose at 5 mm depth, which can be used to prescribe dose on one site or both sites. The mapping functions make it more flexible not only for the nine dose levels, basically any dose from one site to its interpolated corresponding dose on the other site. The double-dose prescription approaches (P2_5mm&Sur and P4_Sur&5mm) in our study are better than the traditional single-site prescription methods (P1_5mm and P3_Sur), making it possible to lower the hot dose on the surface and improve the cold dose at 5 mm depth, which improves the mean dose in general.
